# SOS-independent prophage induction pathways and their contributions to antibiotic resistance gene dissemination: advances and controversies

**DOI:** 10.3389/fmicb.2026.1972752

**Published:** 2026-10-22

**Authors:** Jinwen Sima, Suling Li, Qingju Bian, Yuanjie Chen, Siyu Ji, Yusi Yan, Zewen Zhao, Anqi Liu, Xianxue Shi

**Affiliations:** 1 North Henan Medical University, Xinxiang, China; 2 Chinese Society of Biotechnology, Beijing, China; 3 Zhejiang Chinese Medical University, Hangzhou, China; 4 Zhengzhou Central Hospital, Zhengzhou, China; 5 Henan Medical University, Xinxiang, China; 6 Jilin University, Changchun, China

**Keywords:** antibiotic resistance genes, horizontal gene transfer, lysogenic switch, quorum sensing, SOS-independent prophage induction, sub-inhibitory antibiotics

## Abstract

Temperate bacteriophages integrate into bacterial chromosomes as prophages, which are widespread across sequenced bacterial genomes and serve as key mobile genetic elements mediating horizontal gene transfer and the global dissemination of antibiotic resistance genes (ARGs). For decades, prophage induction has been thought to rely almost entirely on the canonical SOS DNA damage response triggered by DNA-damaging agents such as fluoroquinolones and mitomycin C; however, accumulating experimental evidence shows that multiple SOS-independent regulatory circuits also drive the lysogeny-to-lysis switch in response to non-DNA-damaging physiological and environmental cues. In this mini-review, we classify SOS-independent prophage induction pathways into five major categories: quorum sensing-mediated regulation, metabolic and oxidative stress-triggered activation, induction by sub-inhibitory concentrations of non-DNA-damaging antibiotics, spontaneous induction driven by stochastic gene expression noise, and modulation by physiological and environmental factors such as bile acids and osmotic stress. We summarize the molecular basis of each pathway and examine available empirical evidence linking these induction routes to phage-mediated ARG dissemination across clinical, intestinal, and environmental microbial ecosystems. We further outline four core unresolved debates in the field: the relative quantitative contribution of SOS-independent versus SOS-dependent induction to resistance spread, the mechanistic classification of sub-inhibitory antibiotic effects, the gap between bioinformatic predictions of prophage-associated ARGs and their functional transmissibility, and the limited taxonomic scope of current mechanistic research. We conclude by discussing implications for antimicrobial resistance stewardship and highlighting priority directions for future quantitative and functional investigations.

## 1 Introduction

Temperate bacteriophages persisting as chromosomally integrated prophages are ubiquitous across nearly all sequenced bacterial genomes, constituting a major class of mobile genetic elements that drive bacterial genome plasticity and adaptive evolution (Zhang S. et al., 2025). Beyond their role as infectious agents, prophages encode a broad repertoire of accessory genes—including virulence factors, metabolic enzymes, and antibiotic resistance genes (ARGs)—that confer fitness advantages to their bacterial hosts (Gabashvili et al., 2022). Upon activation of the lytic cycle, induced prophages excise from the host chromosome, replicate, and release progeny particles capable of transferring genetic material to susceptible bacteria via specialized or generalized transduction. This phage-mediated horizontal gene transfer (HGT) is now recognized as a key mechanism underlying the global dissemination of antimicrobial resistance (AMR), contributing to the emergence of multidrug-resistant pathogens in both clinical and environmental settings (Anastassopoulou et al., 2025; Shen et al., 2026).

For over half a century, the SOS DNA damage response has been established as the canonical pathway governing prophage induction. In this classical model, DNA double-strand breaks generated by agents such as fluoroquinolones or mitomycin C lead to the accumulation of single-stranded DNA, which activates the *RecA* co-protease to trigger autocleavage of the bacterial *LexA* repressor and phage-encoded CI repressors, thereby de-repressing lytic gene expression (Nair et al., 2025). This paradigm has dominated research in the field, and investigations into antibiotic-driven prophage activation have long centered on DNA-damaging antimicrobials acting through the SOS cascade (Zhang et al., 2022). As a result, the contribution of non-SOS induction mechanisms to phage ecology and AMR spread has been historically underappreciated.

Accumulating evidence from the past 5 years, however, has revealed a diverse and functionally heterogeneous set of SOS-independent pathways that regulate prophage induction in response to a wide range of physiological and environmental signals. Quorum sensing (QS) systems, which coordinate bacterial population behavior via diffusible signaling molecules, directly modulate the lysogeny-lysis switch in multiple phage-host systems: both host-encoded QS regulators and phage-carried QS receptors can initiate lytic replication independently of DNA damage, timing phage release to coincide with high host cell density for optimal transmission (Shang et al., 2025; Santoriello and Bassler, 2026). Metabolic and oxidative stressors also trigger prophage activation through dedicated, *RecA*-independent regulatory circuits: for instance, redox-active bacterial metabolites such as pyocyanin induce prophage-selective activation in *Staphylococcus aureus* via a high-level oxidative stress (HiLOS) response that does not engage the SOS pathway (Jancheva et al., 2025; Jancheva and Böttcher, 2021). Even antibiotics without DNA-damaging activity—including β-lactams, macrolides, and tetracyclines—can promote prophage induction at sub-inhibitory concentrations, through mechanisms involving ribosomal stress, cell envelope perturbation, or metabolic dysregulation rather than canonical SOS signaling (Yang et al., 2026). Compounding these environmentally triggered pathways is low-frequency spontaneous induction, a universal feature of lysogenic populations driven by stochastic fluctuations in repressor protein expression, which occurs entirely independently of exogenous stress (Zhang S. et al., 2025).

Despite the growing number of mechanistic reports, four critical gaps limit our understanding of SOS-independent prophage induction and its impact on AMR. First, non-SOS induction pathways have been described across disparate bacterial and phage taxa, with no unified classification of their molecular logic, evolutionary conservation, or environmental prevalence. Second, the quantitative contribution of SOS-independent pathways to phage-mediated ARG dissemination remains unresolved: while SOS-dependent induction typically produces higher phage titers, non-SOS pathways may operate continuously under physiological conditions where DNA damage is rare, potentially driving greater cumulative ARG transfer over time (Joglekar et al., 2023). Third, core controversies remain unaddressed: there is ongoing debate over whether bioinformatic predictions of prophage-associated ARGs overestimate their functional transmissibility, and the extent to which non-SOS induction contributes to resistance spread in clinical microbiomes remains unclear (Chang et al., 2025).

This narrative mini-review synthesizes peer-reviewed literature retrieved from PubMed, Web of Science, and Scopus databases. Primary search terms included combinations of “SOS-independent prophage induction”, “lysogeny-lysis switch”, “prophage activation”, “antibiotic resistance genes”, “horizontal gene transfer”, “quorum sensing prophage”, “metabolic stress prophage induction”, “sub-inhibitory antibiotics prophage”, and “spontaneous prophage induction”. We restricted the search to English-language publications published between 2000 and 2026, with priority given to original experimental studies from the past 10 years. Additional studies were identified via backward citation chasing from key reviews and landmark primary articles. Studies were included if they reported mechanistic or quantitative data on prophage induction pathways independent of RecA/LexA-mediated SOS signaling, or on phage-mediated ARG transduction associated with non-SOS triggers. This is not a systematic review; studies were selected to represent the breadth of mechanistic diversity and key controversies in the field, rather than to achieve exhaustive systematic coverage.

This Mini Review provides a critical, focused synthesis of SOS-independent prophage induction mechanisms and their roles in ARG dissemination. We systematically categorize the major classes of non-SOS pathways, detail their underlying molecular basis, and evaluate the empirical evidence linking each pathway to the horizontal transfer of resistance determinants. We further delineate key unresolved controversies in the field, identify priority knowledge gaps, and discuss the implications of these underrecognized induction pathways for clinical AMR stewardship and the development of interventions to curb phage-driven resistance spread.

## 2 SOS-independent prophage induction: molecular mechanisms

The canonical SOS DNA damage response has served as the central paradigm for prophage induction for more than 50 years, but a growing body of work over the past decade reveals that lysogenic phages employ a far broader repertoire of regulatory circuits to initiate lytic replication. These SOS-independent pathways respond to diverse physiological and environmental cues—from population density to metabolic state and cell envelope stress—none of which involve DNA strand breaks or *RecA*-mediated *LexA* cleavage (Zhou and Gottesman, 1998). The molecular architecture of these SOS-independent regulatory circuits and their downstream roles in phage-mediated ARG horizontal transfer are systematically illustrated in Figure 1. Below we systematically outline the major classes of SOS-independent induction mechanisms and their molecular basis. In the following sections, we describe the molecular basis of each pathway and evaluate the strength of evidence for SOS independence using standardized criteria defined in Section 4.2; evidence for downstream ARG dissemination is evaluated separately in Section 3 using a hierarchical functional framework.

**Figure 1:**
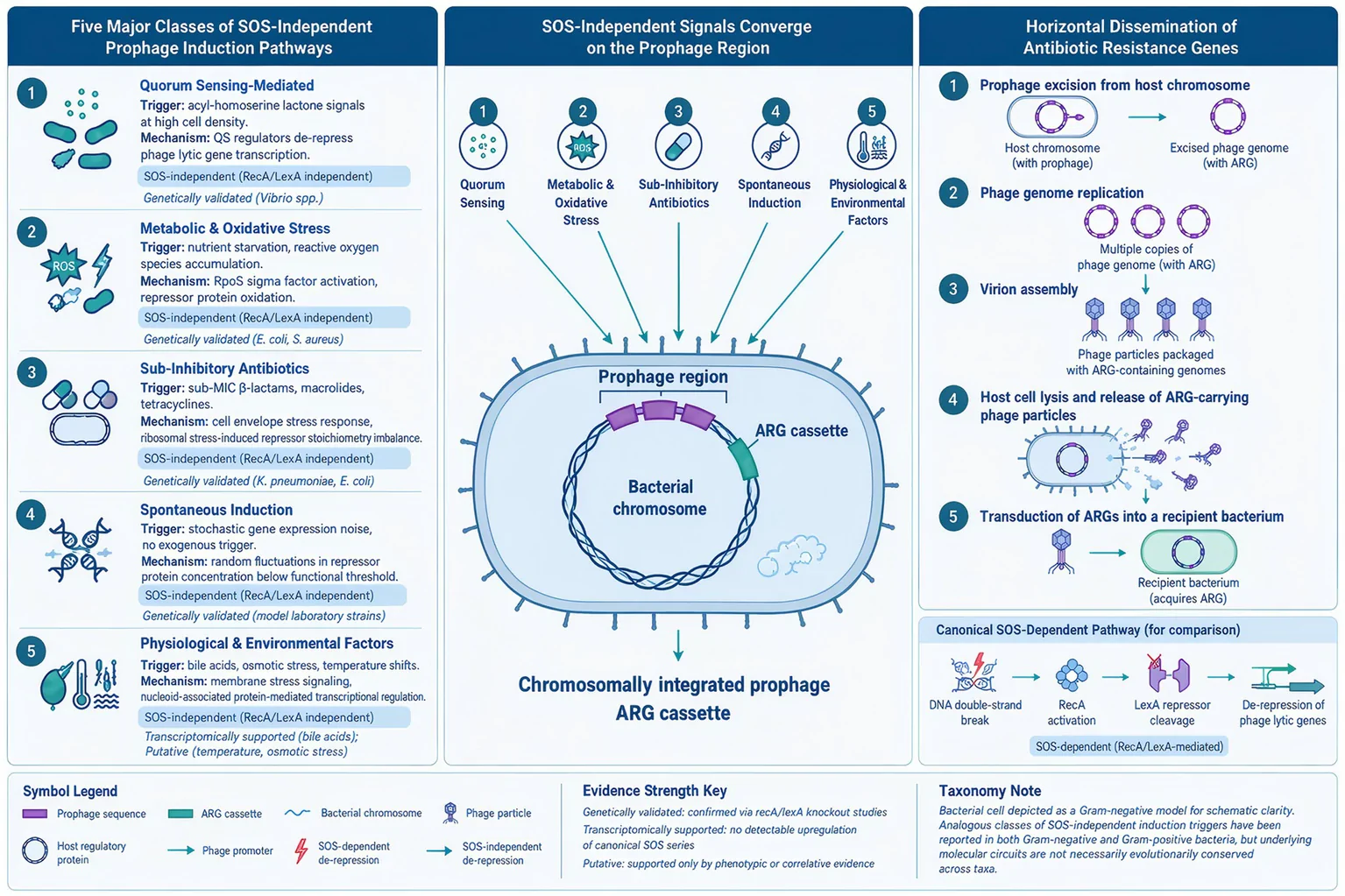
Schematic overview of SOS-independent prophage induction pathways and their contributions to horizontal dissemination of antibiotic resistance genes. *Left*: Five major classes of SOS-independent prophage induction triggers and their corresponding molecular regulatory circuits: (1) quorum sensing-mediated induction via acyl-homoserine lactone signals and quorum sensing (QS) regulators; (2) metabolic and oxidative stress triggered by nutrient starvation and reactive oxygen species via the RpoS (σ^S^) pathway; (3) induction by sub-inhibitory concentrations of non-DNA-damaging antibiotics (β-lactams, macrolides, tetracyclines) via cell envelope stress and ribosomal stress responses; (4) spontaneous induction driven by stochastic gene expression noise; (5) physiological and environmental factors including bile acids, osmotic stress and temperature shifts, mediated by membrane stress signaling and nucleoid-associated proteins. All five pathways operate independently of the RecA/LexA-dependent SOS DNA damage response. Evidence strength for SOS independence is annotated for each class following standardized operational criteria: genetically validated (confirmed via recA/lexA knockout studies), transcriptomically supported (no detectable upregulation of canonical SOS genes), and putative (supported only by phenotypic or correlative evidence). *Center*: A Gram-negative bacterial cell carrying chromosomally integrated prophage sequences and an associated antibiotic resistance gene (ARG) cassette, which serves as the substrate for induction. Signals from all five induction pathways converge on the prophage region to initiate lytic activation. *Right*: The stepwise process of phage-mediated ARG dissemination, including prophage excision, genome replication, virion assembly, host cell lysis and release of ARG-carrying phage particles, and subsequent transduction of ARGs into recipient bacteria. The canonical SOS DNA damage pathway is included at the bottom right as a reference for comparison with non-SOS induction circuits. The bacterial cell is depicted as a Gram-negative model for schematic clarity. Analogous classes of SOS-independent induction triggers have been reported in both Gram-negative and Gram-positive bacteria, but the underlying molecular circuits are not necessarily evolutionarily conserved across taxa. Key examples in Gram-positive species include oxidative stress induction in *Staphylococcus aureus* and *Lactococcus lactis*, and macrolide-induced induction in *Streptococcus pneumoniae*.

### 2.1 Quorum sensing-mediated induction

Quorum sensing (QS) systems coordinate bacterial population-level behaviors via diffusible signaling molecules, and temperate phages have repeatedly co-opted these pathways to tune their lysogeny-lysis switch to host cell density. In this framework, phage induction is driven not by cellular damage, but by the detection of a sufficiently large host population, which maximizes the likelihood that progeny virions will encounter susceptible neighboring cells (Al-Anany et al., 2024).

Two distinct mechanistic strategies have been characterized. First, host-encoded QS regulators can directly bind phage promoter regions to modulate repressor transcription (Dougherty et al., 2023). In *Vibrio cholerae*, for example, the master QS regulator *HapR* accumulates at high cell density and represses transcription of the CTXφ prophage repressor rstR, thereby de-repressing lytic gene expression and promoting phage production. Genetic ablation of HapR abolishes CTXφ induction in dense cultures, while deletion of recA does not eliminate this effect, demonstrating that this pathway is genetically demonstrated SOS-independent (Henrot and Petit, 2022). Second, many phages carry their own QS receptor homologs, enabling them to directly sense extracellular signals without relying on host regulatory cascades. A well-documented example is the *Vibrio anguillarum* phage ΦH20, which encodes a LuxR-type receptor that binds host-produced acyl-homoserine lactone signals and activates lytic replication at threshold concentrations. This phage-encoded QS module has been functionally validated *in vitro*, and bioinformatic surveys identify phylogenetically widespread homologs, suggesting a convergent strategy for density-dependent lifecycle control (Silpe et al., 2022).

In *Pseudomonas aeruginosa*, the Las and Rhl QS systems have been shown to modulate prophage induction dynamics during biofilm growth. Disruption of lasI significantly reduces spontaneous prophage release from biofilm populations, indicating that host QS signaling supports baseline phage production in structured communities; however, the direct molecular link between QS regulators and phage promoter regions remains to be characterized in this system (Giallonardi et al., 2023). Similarly, in *Staphylococcus aureus*, the Agr quorum sensing system is associated with induction of several immune evasion-associated prophages, linking phage activation to bacterial population density during infection. This association is supported by phenotypic data from Agr mutant strains, and these systems are currently putatively SOS-independent pending genetic validation at the molecular level (Hatoum-Aslan, 2021). Collectively, these findings indicate that QS-mediated prophage regulation is not restricted to Vibrio species, and has been documented in phylogenetically diverse pathogenic bacterial lineages. The extent of evolutionary conservation of the underlying molecular circuits across taxa remains to be systematically determined (Bernard et al., 2023).

### 2.2 Metabolic and oxidative stress-triggered induction

Bacteria routinely encounter metabolic perturbations such as nutrient limitation, redox imbalance, and respiratory stress, and these conditions can trigger prophage activation through dedicated regulatory circuits that operate independently of DNA damage (Oh et al., 2019a).

Stationary-phase nutrient starvation is a well-characterized trigger of SOS-independent prophage induction. In *Escherichia coli*, the stationary-phase sigma factor *RpoS* (σ^S^) mediates prophage activation during carbon limitation by directly activating transcription of phage lytic genes, and this process remains fully functional in recA-deficient strains. Mechanistically, RpoS is proposed to bind conserved promoter elements upstream of the phage cro homolog in *λ*-like phages, bypassing CI repressor-mediated silencing that dominates exponential growth. This model is supported by genetic epistasis data in recA-deficient strains, but direct biochemical evidence of promoter binding remains limited for many phage-host pairs (Rozanov et al., 1998). A 2023 study reported that the dietary carbohydrate D-xylose induces prophage production in intestinal *E. coli*, an effect associated with metabolic shifts and RpoS upregulation; formal demonstration of RpoS dependence via knockout studies remains to be provided (Hu et al., 2023).

Oxidative stress represents another major trigger of non-SOS prophage induction. In *S. aureus*, the pyocyanin-induced oxidative stress pathway does not require RecA or LexA and does not upregulate canonical SOS genes such as recN and uvrA, confirming its independence from the classical DNA damage cascade (Jancheva and Böttcher, 2021). Additional work has shown that hydrogen peroxide exposure can induce prophage activation in *Lactococcus lactis* through oxidation of the phage CI repressor, a mechanism that also does not involve RecA activation (Ju et al., 2025). In gut anaerobic environments, metabolic shifts in short-chain fatty acid production also alter prophage induction profiles in commensal *Bacteroides* species, with no associated SOS response signature detected in metatranscriptomic data (Oh et al., 2019b). Not all pathways described above have been formally validated as RecA-independent via genetic knockout studies; some are supported by indirect evidence such as absence of SOS gene upregulation or phenotypic correlation with metabolic state.

### 2.3 Prophage induction by sub-inhibitory antibiotics via non-DNA-damaging pathways

It was long assumed that antibiotic-driven prophage induction was restricted to DNA-damaging agents such as fluoroquinolones that act through the SOS pathway (Nair et al., 2025). However, recent work demonstrates that multiple classes of non-DNA-damaging antibiotics also induce prophage activation at sub-inhibitory concentrations, via mechanisms targeting ribosomal function, cell envelope integrity, or central metabolism (Yang et al., 2026).

β-lactam antibiotics, which inhibit cell wall synthesis, trigger prophage induction in multiple Gram-negative pathogens via cell envelope stress responses rather than DNA damage. In *Klebsiella pneumoniae*, sub-minimum inhibitory concentration (sub-MIC) ceftazidime induces prophage release via cell envelope stress responses. This effect is preserved in lexA mutant strains incapable of mounting a canonical SOS response, providing direct genetic evidence for SOS independence (Al-Anany et al., 2024). Preliminary evidence also suggests similar sub-MIC β-lactam induction of prophages in carbapenem-resistant *Acinetobacter baumannii*, putatively mediated by envelope stress pathways in the absence of a canonical SOS response (Hare et al., 2014).

Similarly, macrolide and tetracycline antibiotics, which inhibit protein synthesis, promote prophage induction by disrupting the stoichiometric balance between phage repressor proteins and their target promoters. Reduced *de novo* repressor synthesis leads to gradual dilution of repressor molecules during cell division, eventually tipping the balance toward lytic replication in the absence of DNA damage (Yang et al., 2026). This mechanism is supported by quantitative measurements of repressor protein dynamics and by retention of induction in recA-deficient *E. coli* strains (Hao et al., 2025). In Gram-positive pathogens such as *Streptococcus pneumoniae,* sub-MIC macrolides (e.g., erythromycin) also promote prophage induction via ribosomal stress and disruption of repressor protein stoichiometry. Notably, pneumococci lack a canonical LexA-mediated SOS response and rely on competence-associated pathways for DNA damage repair, such that macrolide-driven prophage activation occurs independently of the classical SOS cascade (Yang et al., 2026; Al-Anany et al., 2024). Critically, these non-DNA-damaging drug classes are among the most widely prescribed antimicrobial agents in clinical practice, suggesting their contribution to phage-mediated resistance spread may be substantially underrecognized (Supina and Dennis, 2025).

For trimethoprim, trimethoprim-induced induction is currently putatively SOS-independent, based on indirect evidence, based on the absence of detectable DNA strand breaks and lack of SOS reporter activation in treated cultures (Zhang L. et al., 2025). These findings indicate that non-SOS prophage induction is a widespread response to antibiotic exposure, not limited to a single drug class (Jaffal et al., 2024).

### 2.4 Spontaneous prophage induction

Spontaneous prophage induction has been detected in nearly all well-characterized lysogenic bacterial laboratory strains, in which a small fraction of cells enters the lytic cycle in the absence of identifiable exogenous stress. This phenomenon is thought to be driven by stochastic fluctuations (gene expression noise) in phage repressor protein concentration, and to occur independently of the SOS pathway, as it persists under non-stress growth conditions and does not require exogenous DNA damage (Dougherty et al., 2023).

Spontaneous induction frequency varies widely across characterized phage-host systems, typically ranging from 10^−6^ to 10^−2^ per cell per generation in laboratory isolates. This range is derived from quantitative bulk culture measurements in model systems such as lambdoid prophages in *E. coli* and staphylococcal prophages, and is determined primarily by the kinetic properties of the phage repressor-operator interaction (Czyz et al., 2001; Utter et al., 2014). The degree to which this range generalizes to uncharacterized environmental lysogens remains unclear.

While per-cell induction rates are low, spontaneous induction can occur continuously under non-stressed baseline growth conditions, though its frequency varies substantially with host strain, prophage identity, growth phase, and environmental context. It is hypothesized to be a major source of free phage particles in stable, homeostatic natural microbial communities. In soil rhizosphere microbiomes, for example, metagenomic and cultivation-based studies of Bradyrhizobium populations indicate that spontaneously induced prophages represent a major component of detectable free phage particles under baseline conditions (Joglekar et al., 2023).

In the human gut microbiome, spontaneous prophage induction is thought to support a baseline of free phage particles that may contribute to steady-state horizontal gene transfer between commensal bacteria, though direct *in vivo* quantification of transduction rates remains limited. Comprehensive reviews of human gut virome surveys consistently indicate that spontaneous prophage release is a ubiquitous feature of healthy adult gut ecosystems (Ogilvie and Jones, 2015). Spontaneous induction also confers fitness benefits to the host population, as released phages kill competing non-lysogenic strains via a process termed lysogen allelopathy (Mahmud et al., 2024). This altruistic behavior is thought to be a major driver maintaining lysogeny in natural bacterial populations (Fatima and Hynes, 2025).

### 2.5 Additional host and environmental drivers

A range of other host-associated and environmental factors have been implicated in modulating prophage induction, with varying levels of evidence for SOS independence.

Among the best-characterized physiological triggers are bile acids in the mammalian gut, which act as potent inducers of prophages in both commensal and pathogenic bacteria. Bile salts disrupt bacterial membrane integrity and trigger intracellular signaling cascades that activate phage lytic genes. Multiple studies have confirmed that bile acid-induced induction occurs independently of DNA damage and does not upregulate canonical SOS response genes; these are transcriptomically supported SOS-independent pathways (Peters et al., 2024). A 2025 study further demonstrated that conjugated bile acids induce species-specific prophage activation in gut Bacteroidetes, shaping phage community dynamics in the distal intestine (Richards et al., 2024).

Other physicochemical factors including temperature shifts, pH fluctuations, and osmotic stress have been proposed to modulate induction by altering repressor protein conformation or host nucleoid-associated protein binding to phage genomes, but their SOS independence has not been uniformly validated across systems (Li et al., 2025). For instance, the nucleoid-associated protein H-NS can silence or activate phage promoters in response to environmental changes, providing a potential layer of SOS-independent regulation. H-NS-mediated silencing of prophage genes is relieved under low-temperature and high-osmolarity conditions, leading to de-repression of lytic genes and phage release. While this pathway does not directly involve *RecA* or *·*, formal genetic validation of SOS independence under these specific conditions remains limited (Schmidt et al., 2024). In *Salmonella enterica*, osmotic stress has been shown to induce prophage activation via the *RpoS* sigma factor pathway, with no evidence of SOS response activation in transcriptomic assays, supporting SOS-independent regulation in this organism (Mayer et al., 2025).

The core features and reported qualitative relative induction magnitudes of all major SOS-independent pathways are summarized in Table 1; the magnitude categories represent approximate qualitative estimates derived from cross-study comparisons, as uniform head-to-head quantification remains unavailable.

**Table 1:** Comparative summary of SOS-independent prophage induction pathways.

Pathway category	Triggering stimuli	Core molecular mechanism	Qualitative relative induction magnitude (vs. MMC SOS)	SOS independence evidence	ARG dissemination evidence	Key references
Quorum sensing-mediated	Acyl-homoserine lactones, autoinducing peptides (high cell density)	QS regulators modulate phage repressor transcription; phage-encoded QS receptors sense host signals	Moderate	Genetically demonstrated (*Vibrio* spp.); putative in other taxa	Phage production demonstrated; indirect metagenomic evidence for ARGs	Shang et al. (2025); Henrot and Petit (2022); Giallonardi et al. (2023)
Metabolic & oxidative stress	Nutrient starvation, stationary phase, redox-active metabolites, ROS	Sigma factor switching (RpoS); repressor oxidation and destabilization	Low to moderate	Genetically demonstrated (*E. coli*, *S. aureus*); transcriptomically supported (gut communities)	Functional transduction demonstrated in gut microcosms	Jancheva and Böttcher (2021); Zhou and Gottesman (1998); Oh et al. (2019b)
Sub-inhibitory non-DNA-damaging antibiotics	β-lactams, macrolides, tetracyclines at sub-MIC	Cell envelope stress activation; repressor stoichiometric imbalance via translation inhibition	Moderate	Genetically demonstrated (*K. pneumoniae*, *E. coli*); transcriptomically supported (*A. baumannii*)	Functional transduction demonstrated (carbapenem, tetracycline resistance genes)	Al-Anany et al. (2024); Hare et al. (2014); Zhang et al. (2025)
Spontaneous induction	Stochastic gene expression noise; no exogenous trigger	Random repressor concentration fluctuations below functional threshold	Very low	Genetically demonstrated (model laboratory strains)	Phage production demonstrated; hypothesized continuous low-level transfer	Joglekar et al. (2023); Dougherty et al. (2023); Czyz et al. (2001)
Physiological & environmental factors	Bile acids, temperature shift, pH change, osmotic stress	Membrane stress signaling; nucleoid protein-mediated transcriptional regulation	Low to moderate	Transcriptomically supported (bile acids); putative (temperature, osmotic stress)	Indirect metagenomic evidence from gut microbiomes	Peters et al. (2024); Richards et al. (2024); Mayer et al. (2025)

Induction magnitude categories are qualitative comparative estimates synthesized across heterogeneous published studies using different phage-host systems, induction assays, and mitomycin C (MMC) concentrations. No uniform head-to-head comparison of all five pathways in a single isogenic experimental system has been performed. Categories are provided only to illustrate general relative differences between pathway classes and should not be interpreted as precise quantitative values.

Evidence strength for SOS independence follows the operational criteria defined in Section 4.2: genetically demonstrated = confirmed via recA/lexA knockout studies; transcriptomically supported = no detectable upregulation of canonical SOS genes; putative = supported only by phenotypic or correlative evidence.

### 2.6 Contribution to antibiotic resistance gene dissemination

To frame the discussion of phage-mediated ARG dissemination, we use a hierarchical evidence framework that distinguishes five sequential levels of experimental demonstration: (1) prophage induction (excision and initiation of lytic gene expression), (2) viable phage production (release of infectious virion particles), (3) ARG packaging (presence of resistance gene sequences within phage particles), (4) successful transduction (stable integration and expression of ARGs in recipient bacteria), (5) phenotypic resistance (conferral of reduced antibiotic susceptibility in transductants). Throughout this section, we explicitly indicate the highest evidence level reached for each reported association between SOS-independent induction and ARG dissemination.

The discovery of diverse SOS-independent induction pathways has fundamentally revised our understanding of the ecological drivers of phage-mediated antibiotic resistance gene (ARG) dissemination (Vu et al., 2024). Historically, research on phage-driven resistance spread focused on SOS-inducing DNA-damaging agents, which generate high phage titers but are relatively rare in natural and clinical environments (Guo et al., 2024). In contrast, SOS-independent pathways are activated by commonplace physiological and environmental signals, suggesting they may drive more sustained, widespread ARG transfer under real-world conditions (Vale et al., 2024).

In non-clinical microbial ecosystems, spontaneously induced and metabolic stress-induced prophages are thought to represent major sources of active phage particles under baseline conditions. In soil microbiomes, cultivation-based and metagenomic data suggest that spontaneous induction accounts for the majority of detectable free phages in the absence of exogenous stressors (evidence level 2: viable phage production) (Joglekar et al., 2023). In the human gut microbiome, baseline prophage release is also widely observed and linked to both spontaneous and metabolic triggers (evidence level 2: viable phage production) (Ogilvie and Jones, 2015). Evidence from aquatic systems remains more limited, with similar patterns proposed but less extensively quantified. Under homeostatic conditions, spontaneously induced prophages are thought to support a baseline of free phage particles (evidence level 2: viable phage production), which may facilitate continuous exchange of ARGs between commensal and pathogenic bacteria, though direct *in vivo* measurement of transduction remains limited (Ogilvie and Jones, 2015). Metabolic fluctuations, such as nutrient availability gradients in the gut lumen, further amplify this baseline transfer by triggering additional rounds of prophage activation via RpoS-dependent and other metabolic pathways (Huang et al., 2025). A review of gut microbiota prophage induction signals indicates that physiological concentrations of bile acids induce prophage activation and promote phage-mediated ARG transfer, an effect that appears to persist independently of canonical SOS signaling (evidence level 3: ARG packaging) (Henrot and Petit, 2022).

In clinical settings, sub-inhibitory concentrations of non-DNA-damaging antibiotics represent a particularly important driver of SOS-independent ARG dissemination (Taylor et al., 2025). Sub-MIC antibiotic concentrations are widespread in patient tissues, hospital wastewater, and agricultural environments, and they have been shown to significantly accelerate phage-mediated ARG transfer (Ma and Zhang, 2025). Studies of clinical *K. pneumoniae* isolates indicate that sub-MIC ceftazidime promotes transduction of bla_KPC_ carbapenemase genes via an SOS-independent envelope stress pathway (evidence level 4: successful transduction), challenging the long-held view that only fluoroquinolones and other DNA-damaging drugs promote phage-driven resistance spread. Similarly, sub-MIC tetracycline has been shown to increase phage-mediated transfer of tetracycline resistance genes in *E. coli* isolates from livestock environments (evidence level 4: successful transduction) (Floccari et al., 2025).

Wastewater treatment plants represent another key environmental reservoir where sub-inhibitory antibiotic concentrations drive prophage induction and ARG dissemination. Metagenomic evidence from human-impacted environments (including wastewater treatment plants) shows that prophage-associated ARGs are consistently enriched under low-level β-lactam selective pressure (evidence level 3: ARG packaging) (Liao et al., 2024). These findings suggest that non-SOS induction pathways contribute substantially to ARG propagation in anthropogenically impacted environments (Shen et al., 2025).

Despite this growing body of evidence, it is important to note that not all induced phage particles mediate functional ARG transfer (Chang et al., 2025). Many prophage-associated ARGs predicted by bioinformatics are transcriptionally silent or located in defective prophage regions incapable of producing infectious virions, and the actual rate of functional ARG transduction via SOS-independent pathways remains a matter of active investigation (Ortiz de Ora et al., 2025). Collectively, these documented cases span evidence levels 2–4: direct functional transduction evidence (level 4) is concentrated in well-controlled clinical isolate systems, while environmental and gut microbiome data remain predominantly at levels 2–3, pending further *in vivo* quantitative validation.

## 3 Controversies and unresolved questions

The field of SOS-independent prophage induction is rapidly evolving, and several core controversies remain unresolved. Addressing these questions is critical for accurately quantifying the impact of these pathways on antimicrobial resistance spread and developing effective mitigation strategies (Liao et al., 2024).

### 3.1 Quantitative contribution relative to canonical SOS induction

A central debate concerns the relative importance of SOS-independent versus SOS-dependent pathways in driving ARG dissemination in natural and clinical settings. It is well established that SOS-dependent induction by strong DNA-damaging agents (e.g., mitomycin C, fluoroquinolones) typically produces much larger burst sizes per induced cell: in model systems such as *λ* phage in *E. coli*, MMC-induced burst sizes are commonly 10–100-fold higher than those of spontaneous or metabolic stress-induced induction (Nair et al., 2025). This has led to the view that SOS-dependent induction remains the dominant driver of acute, high-magnitude ARG transfer events.

The opposing perspective emphasizes that SOS-inducing DNA damage events are rare and transient in most natural and clinical environments, whereas SOS-independent pathways operate continuously under baseline physiological conditions, potentially resulting in greater cumulative ARG transfer over longer time scales.

Rather than a binary opposition, the relative contribution of each pathway class to total ARG dissemination is likely determined by the product of multiple interconnected factors: 1. Induction frequency: how often the trigger occurs in a given environment; 2. Burst size: number of phage particles produced per induced cell; 3. ARG carriage rate: fraction of induced phages that carry functional ARG cassettes; 4. Transduction efficiency: probability that an ARG-carrying phage successfully infects a recipient and stably integrates the resistance gene; 5. Ecological persistence: survival time of free phage particles in the environment.

Currently, quantitative data from complex microbial communities are scarce, and no study has directly compared the total ARG transfer burden attributable to each pathway class in a clinically relevant ecosystem (Stopnisek et al., 2025).

### 3.2 Mechanistic classification of sub-inhibitory antibiotic effects

There is ongoing disagreement over whether prophage induction by sub-inhibitory antibiotics truly represents SOS-independent signaling, or whether it reflects low-level DNA damage that falls below conventional detection thresholds. Some researchers argue that even non-DNA-damaging antibiotics can indirectly cause oxidative stress and DNA lesions at sub-MIC concentrations, leading to weak, undetectable SOS activation that nonetheless drives prophage induction (Zhang et al., 2022). However, multiple studies have demonstrated that antibiotic-induced prophage activation remains fully intact in *ΔrecA and ΔlexA* mutant strains incapable of mounting an SOS response, providing strong genetic evidence for true SOS-independent mechanisms (Yang et al., 2026). This controversy is compounded by the fact that mechanisms vary across different antibiotic classes and phage-host systems, making broad generalizations difficult (Iqbal and Begum, 2024).

To advance this debate, we propose a hierarchical set of operational criteria for classifying a prophage induction pathway as SOS-independent, ordered by strength of evidence: 1. Strong genetic evidence: Induction persists at equivalent levels in isogenic *ΔrecA* or non-cleavable lexA mutant strains, relative to wild-type controls. This eliminates the possibility of *RecA/LexA*-dependent signaling; 2. Strong transcriptomic evidence: Induction occurs without detectable upregulation of canonical SOS response genes (e.g., *recA*, *lexA*, *recN*, *uvrA*) as measured by qRT-PCR or RNA-seq, and in the absence of detectable DNA strand breaks; 3. Weak phenotypic evidence: Induction occurs in response to a stimulus not known to cause DNA damage, but genetic or transcriptomic validation of SOS independence is absent.

Pathways that meet only criterion 3 should be classified as putatively SOS-independent until further validation. Applying this standardized framework across studies would greatly improve comparability and help resolve current disagreements.

### 3.3 Discrepancy between bioinformatic predictions and functional transmissibility

A third major controversy surrounds the actual clinical relevance of prophage-mediated ARG transfer. Bioinformatic analyses of bacterial genomes and metagenomes frequently identify ARGs within predicted prophage regions, leading to claims that phages are major vectors of global resistance dissemination (Gabashvili et al., 2022). However, experimental validation studies have found that the majority of predicted prophage-associated ARGs are not functionally transduced by active phage particles, and many reside in defective prophage regions incapable of producing infectious virions (Chang et al., 2025). This discrepancy has led to a polarized debate: one side argues that bioinformatic surveys drastically overestimate the role of phages in antimicrobial resistance spread, while the other maintains that low-frequency transduction events, though difficult to detect experimentally, are ecologically significant at the population level (Giallonardi et al., 2023). Resolving this controversy requires standardized functional assays and quantitative measurements of *in situ* transduction rates (Ortiz de Ora et al., 2025).

### 3.4 Evolutionary conservation across bacterial taxa

Most research on SOS-independent induction pathways has been conducted in Gram-negative model organisms such as *E. coli*, *V. cholerae*, and *P. aeruginosa* (Zhou and Gottesman, 1998). Far less is known about these pathways in Gram-positive bacteria, strict anaerobes, and other understudied taxa. For example, while spontaneous induction appears to be universal, it remains unclear whether QS-mediated and metabolic stress-mediated induction pathways are conserved across distantly related bacterial lineages, or whether they evolved independently in specific phage-host pairs (Bernard et al., 2023). This taxonomic bias limits our ability to generalize findings across microbial ecosystems and assess the global impact of SOS-independent pathways on antimicrobial resistance dissemination (Richards et al., 2024).

## 4 Conclusion

For over half a century, the SOS DNA damage response has been regarded as the canonical pathway governing prophage induction. However, accumulating evidence demonstrates that a diverse set of SOS-independent pathways—modulated by quorum sensing, metabolic state, cell envelope stress, stochastic gene expression noise, and environmental cues—jointly regulate the lysogeny-lysis switch and modulate the kinetics and magnitude of phage-mediated antibiotic resistance gene (ARG) dissemination. Together, these pathways allow prophages to sense and integrate a broad spectrum of host physiological and environmental signals beyond DNA strand breaks, indicating that prophage lifestyle switching reflects a more integrated adaptive response than the classical DNA damage-centric paradigm.

Addressing the four core unresolved controversies outlined above will require targeted methodological and experimental advances. First, quantitative *in situ* approaches with single-virion or single-cell resolution are required to directly quantify the relative contributions of SOS-dependent and SOS-independent induction pathways to ARG transfer in complex microbial communities, rather than relying on indirect bioinformatic inference. Second, mechanistic investigations must be expanded beyond the current predominance of Gram-negative model organisms to include Gram-positive pathogens, obligate anaerobes, and environmental isolates, to rigorously assess the evolutionary conservation and global distribution of these induction pathways. Finally, standardized functional assays for prophage-mediated ARG transduction are necessary to resolve the discrepancy between bioinformatic predictions of prophage-associated ARGs and their experimentally verified transmissibility.

From an antimicrobial stewardship perspective, the ubiquity of SOS-independent induction triggers in clinical, intestinal, and environmental ecosystems suggests that these pathways are likely to make a substantially underappreciated contribution to phage-mediated antimicrobial resistance (AMR) dissemination. Targeting these SOS-independent regulatory circuits—for instance, through quorum sensing inhibition or modulation of cell envelope stress pathways—represents a promising but as-yet unvalidated complementary strategy to mitigate phage-driven ARG spread. Additionally, optimizing antibiotic dosing and administration strategies to reduce sub-minimum inhibitory concentration (sub-MIC) antibiotic exposure could help limit inadvertent prophage induction and subsequent resistance gene transfer in clinical and agricultural settings. A comprehensive mechanistic understanding of the full prophage induction regulatory network is a prerequisite for developing targeted interventions to constrain phage-facilitated AMR dissemination.
